# Application of Forensic DNA Phenotyping for Prediction of Eye, Hair and Skin Colour in Highly Decomposed Bodies

**DOI:** 10.3390/healthcare11050647

**Published:** 2023-02-23

**Authors:** Matteo Fabbri, Letizia Alfieri, Leila Mazdai, Paolo Frisoni, Rosa Maria Gaudio, Margherita Neri

**Affiliations:** 1 Department of Medical Sciences, University of Ferrara, 44121 Ferrara, Italy; 2 Department of Neuroscience and Rehabilitation, University of Ferrara, 44121 Ferrara, Italy; 3 Unit of Legal Medicine, Azienda USL di Ferrara, 44121 Ferrara, Italy; 4 Department of Translational Medicine, University of Ferrara, 44121 Ferrara, Italy

**Keywords:** forensic DNA phenotyping, predictive DNA analysis, HIrisPlex-S, eye colour, skin colour, hair colour, identification

## Abstract

In the last few years, predicting externally visible characteristics (EVCs) by adopting informative DNA molecular markers has become a method in forensic genetics that has increased its value, giving rise to an interesting field called “Forensic DNA Phenotyping” (FDP). The most meaningful forensic applications of EVCs prediction are those in which, having only a DNA sample isolated from highly decomposed remains, it is essential to reconstruct the physical appearance of a person. Through this approach, we set out to evaluate 20 skeletal remains of Italian provenance in order to associate them with as many cases of missing persons as possible. To achieve the intended goal, in this work we applied the HIrisPlex-S multiplex system through the conventional short tandem repeats (STR) method to confirm the expected identity of subjects by evaluating phenotypic features. To investigate the reliability and accuracy of the DNA-based EVCs prediction, pictures of the cases were compared as they were available to researchers. Results showed an overall prediction accuracy greater than 90% for all three phenotypic features—iris, hair, and skin colour—at a probability threshold of 0.7. The experimental analysis showed inconclusive results in only two cases; this is probably due to the characteristics of subjects who had an intermediate eye and hair colour, for which the DNA-based system needs to improve the prediction accuracy.

## 1. Introduction

Many forensic techniques are used today to identify a human corpse, mainly relating to the conditions of the remains. According to the Interpol Protocol [1], the four post-mortem recognised methods for corpse identification are finding of physical indications [2], matching of fingerprints [3], when pre-mortem inked prints are available, dental examination [4], when pre-mortem dental radiographs are available, and DNA analysis [5].

Poor conditions of preservation of the remains, due to an advanced state of decomposition, intervention of physical/chemical elements or micro/macro fauna, often result in high fragmentation, degradation, and mixing of biological samples [6,7,8].

In these cases, it is customary to resort to the evaluation of bone remains with methods of forensic anthropology. Forensic anthropology can be defined as the study of the morphometric characteristics of bone tissue. This approach is inescapably useful in the first phase of the identification process, such as the collection of personal identification characteristics (such as gender, age, height, ethnic group, body conformation, etc.), especially for bodies in an advanced state of decomposition. However, for what concerns the identification processes, the data that can be obtained through anthropological investigations are often insufficient [9,10,11]. It seems therefore fundamental that these data have to be completed by adding other individual characteristics such as the chromatic somatic characters (skin pigmentation, eyes, and hair), iatrogenic or pathological outcomes (prostheses, metal plates, metal stitches, etc.), and other signs (tattoos, piercings, etc.) [12,13]. Especially in those corpses found in a scant state of preservation, however, these characters are not detectable, and the anthropological investigation does not look capable of providing a sufficient probabilistic value, such as to ascertain the identity of the subject, as it can only allow an evaluation in terms of concordance between the data of ante and post-mortem.

For this reason, DNA profiling is the most widespread and reliable method of personal identification in forensics when this must take place on highly degraded human remains [14] and no dental records are available or the concordance of the fingerprints cannot be determined [15,16].

For genetic identification of remains, the Interpol DNA-specific recommendations for Disaster Victim Identification (DVI) are also proposed by the ISFG membership for single-case identification in order to provide an internationally standardised method. The timing and proper storage of post-mortem samples affect the success rates of DNA typing. During the autopsy or external examination, the forensic geneticist, or a pathologist with a background in forensic genetics, should ideally be available for consultation during DNA sample collection.

However, genetic identification linked to DNA profiling still poses some challenges today. One method to redesign STR primers that generate shorter amplicons is to improve the success rate of degraded DNA. During collaborative European exercises, the employment of several STR systems with size-reduced fragments gave positive results when applied to artificially degraded DNA [17,18]. In cases where the starting material was of a reduced quantity, as in contact traces derived from the victims’ personal items, the available DNA extracted reflects the low level of material for genetic analysis. In other cases, relatives’ biological specimens may be unavailable for genetic analysis and comparison as well [19]. These are the major limitations that confront forensic DNA (fDNA), as well as in an anthropological context where samples on which to apply molecular investigations are often both inadequate and unrepresentative [20]. In these cases—especially on anthropological remains—it is possible to refer to mitochondrial DNA (mtDNA), which, by an intrinsic property of the organelle, allows for greater protection of the nucleic acid and consequently better preservation [21]. In addition, within a eukaryotic cell, mtDNA is present in significantly more copies than nuclear DNA (nuDNA), which is present in single units [22,23]. However, mtDNA does not allow a true comparison investigation; this is because it is inherited exclusively matrilineally and, for this reason, is identical in individuals descended from the same matrilineal line [24]. Therefore, it is intuitive to understand why a mitochondrial DNA match cannot lead to absolute identification because, for example, comparison references must come from maternal relatives since mtDNA is inherited exclusively from the maternal parent, and unfortunately, this is not always practicable [25].

The direct consequence of degenerative processes occurring in the post-mortem period is cellular breakdown, which is certainly promoted by extreme environmental conditions (e.g., high temperatures and oxygen deficiency) [26]. All of this results in a completely random degradation of the nuDNA molecule, promoted and facilitated through the action of intracellular enzymes (e.g., nuclease, lipase, and protease) that are released due to membrane failure [27]. Comparatively few environmental conditions provide better preservation and protection of biological matrices from degradation. The more stabilised DNA will be more likely to be found in skeletal tissue than in the soft tissues of the organism. Indeed, the gold standard in these situations—anthropological and forensic—is represented by the bone matrix [28]. As per forensic practice, depending on corpse preservation, different tissue types should be collected, as suggested by Prinz, Carracedo et al. [29]. In particular, for very decomposed or segmented remains, it would be preferable to collect fragments of any available bone or healthy teeth to be used as a substrate for DNA extraction. Cancellous bone can be rich in DNA, but its preservation is not reliable, so in these cases, dense cortical bone should always be the first choice, preferably taken from long bones of the lower limbs [29].

Given this premise, in those cases where standard forensic DNA analysis based on short tandem repeats (STRs) or other types of polymorphic DNA markers such as single nucleotide polymorphisms (SNPs) fails to identify the donor of a human biological trace found at a crime scene or on an unidentified corpse via comparative DNA profiling, alternative approaches are needed [30,31,32].

Forensic DNA phenotyping (FDP) analysis, as a matter of fact, could be crucial in the forensic field in the event that standard DNA analysis is not possible in the absence of a comparison sample [19]. The aim of phenotype studies, through the analysis of DNA sequences, is to predict external somatic traits by processing biological material from unknown corpses or from identified biological traces found during crime scene recognition. Throughout the years, the evolution of this technology has allowed the development of a promising technique due to the evidence it can produce from biological tests [33]. FDP can lead to the prediction of the visible external characteristics of a subject, useful for its identification, starting either from traces of DNA detected at the scene of a crime or from DNA extracted from human remains, even those that are highly decomposed. This can be very useful in identifying intent in cases of missing persons or in the need to use alternative methods for the identification of victims of a mass disaster, especially where ante-mortem reference specimens and/or known relatives are unobtainable [33].

Although external visible characteristics (EVC) are considered complex traits, in which multiple genes contribute to the phenotype along with environmental factors, human pigmentation traits are found to be the least complicated of the EVCs, with only a few genes providing the vast amount of phenotypic information. For this reason, currently, the understanding of the genetic basis of chromatic traits is more studied and known than for other EVCs. The problem with extremely complex genetic characteristics, as extensively studied for many common diseases, is that any single gene contributes only a small portion of the phenotypic variance, and only the combination of a large number of genes accounts for the inherited trait [34].

Precisely in this study, we applied a DNA-based method for the prediction of eye, hair, and skin colour on 20 identification cases dealing with skeletal remains previously identified by the laboratory using conventional STRs analysis. Bone samples, taken from 20 different missing subjects, were chosen for the experimental application because DNA showed a high degree of degradation, resulting in partial STR profiles. The cases have been chosen because pictures of the victims were available as a reference document; photographs were used in order to test and verify the fidelity and accuracy of the DNA multiplex reaction.

Among the currently available statistical models, we choose to apply the HIrisPlex-S system (HIrisPlex-S typing software, open source software available at https://hirisplex.erasmusmc.nl/, accessed on 17 February 2023, Copyright of the Department of Genetic Identification of Erasmus MC, Rotterdam, The Netherlands), which consists of 41 DNA variants: 24 included in the HIrisPlex assay and 17 variants investigated with another analysis. Both multiplex assays can be carried out even with a minimum amount of DNA of 63 pg [35]. The analysis stipulates that subsequently the obtained genotype data are entered into a tool published on the site https://HIrisPlex.erasmusmc.nl/, accessed on 17 February 2023, which is useful for evaluating prediction probabilities for 3 iris colours, 4 hair colours, and 5 skin colour categories [36].

Moreover, this system has been preferred because it has been shown to be experimentally applied to naturally degraded genetic material. In fact, the HIrisPlex system was applied by J. Draus-Barini et al. to DNA samples extracted from old and ancient tissues, demonstrating its suitability in deteriorated DNA analysis. Their research gave promising results on very ancient and therefore very fragmented genetic material, revealing that out of the 26 DNA extracts from bones and teeth between 1 and about 800 years of post-mortem age, 23 yielded a complete 24 SNP profile [37]. Furthermore, in 2014, King, T. et al. applied the HIrisPlex system to even older human tissues, in particular skeletal remains dating back to 1400 AD, leading researchers to the prediction of somatic characteristics (eye and hair colour) by King Richard III of England (1452–1485 AD). The predicted features appeared consistent with the known portraits of the sovereign [38].

Furthermore, the HIrisPlex-S system is forensically endorsed, as forensic validation tests have been carried out and published for both multiplexes, in accordance with the guidelines of the Scientific Working Group on DNA Analysis Methods (SWGDAM) [36,37,38,39].

## 2. Materials and Methods

### 2.1. Sample Selection

The preliminary step that preceded the effective analytical steps involved the evaluation of bone matrix samples from the 20 subjects under study. A total of 14 femurs and 6 tibias were selected, respectively, in case there were bodies for whom visual identification was impossible, due to the very poor conservation state of the corpse. Indeed, cases for the present study have been selected from those subjected to advanced decomposition or massive damage that occurred due to the particular circumstances of death or due to attempts to conceal or destroy the body. Specifically, corpses prone to thermal or mechanical damage (e.g., combustion, explosion, dismemberment) were included in the study. The post-mortem interval (PMI) of the cadavers included in the study ranged from a few days, for those cases in which massive body alteration had occurred secondary to the peculiar causes of death (explosion, charring), up to about 30 years in cases of exhumation of skeletal remains. The cases were selected from those received, due to the need for multidisciplinary forensic analysis, at the Laboratory of Genetics of the Unit of Legal Medicine of the University of Ferrara. For each case, a complete external inspection, autopsy (if possible due to the presence of residual soft tissues), anthropological evaluation, and identification process were carried out.

### 2.2. DNA Extraction and Phenotyping

In all cases, a standard forensic DNA analysis based on short tandem repeats (STR) was performed. For each case, it has been possible to reach a correct personal identification by comparing the data obtained from the cadaver analysis, with data stored in the register of missing persons or with investigative files. As a result of the personal identification, photographic reproductions of the face of the missing person were available in order to trace the externally visible characteristics. The photographs were extracted from the files provided by the prosecutor’s office, from the obituary pages of the local press, or from websites specialised in the search for missing persons. More details about the samples tested are summarised in Table 1.

**Figure 1:**
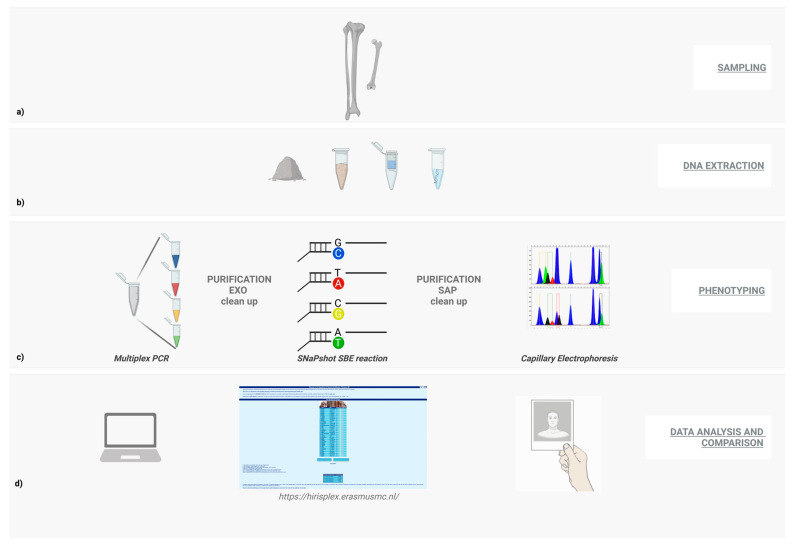
Illustration of the workflow for multiplex PCR phenotyping. Outlining of the main work steps: (**a**) sampling, (**b**) gDNA extraction, (**c**) multiplex PCR, purification of amplicons with exonuclease, SBE reactions, enzymatic purification with SAP, capillary electrophoresis, (**d**) integration of genetic data in the on-line table to obtain phenotypic results, and finally, comparison of these with the photograms of the individuals.

After appropriate and careful sampling was performed, the analytical laboratory steps were proceeded with, which are outlined in Figure 1. For every corpse, one sample of bone powder was collected (femur or tibia as reported in Table 1), except for samples named I7 and I10, where two bone powder samples were collected (femur sample I7; tibia sample I10) due to the high degree of degradation shown by these specimens. For the phenotyping investigation, we decided to apply the HIrisPlex-S system following the recommendations available in the literature [36,37,38,39,40]. We applied the prediction system through the combined approach of multiplex PCR with SNaPshot single base extension (SBE) reactions to analyse multiple SNP in samples with different levels of gDNA degradation [41,42].

For nucleic acid extraction, all samples of the bone matrix were treated appropriately. First, each bone sample was chemically treated, using diluted bleach, and then irradiated with UV light for 30 min, before being powdered as much as possible.

Then, after the appropriate powdering of the bone matrix has been achieved, genomic DNA (gDNA) extraction can proceed. The procedure is performed using an input of 0.5 g of pulverised bone material as a starting point. The gDNA was extracted from the samples under study using the QIAamp DNA Investigator Kit (Qiagen^®^ purchased at QIAGEN S.r.l.—Via Filippo Sassetti n. 16, 20124 Milano, Italy) according to the manufacturer’s guidelines. The extracted and purified nucleic acid obtained was quantified by following the instructions of the Investigator Quantiplex Hyres Kit (Qiagen^®^ purchased at QIAGEN S.r.l.—Via Filippo Sassetti n. 16, 20124 Milano, Italy).

Initially, short adjacent regions of marker SNPs are amplified in a multiplex PCR reaction [43] with a total volume of 10 µL. For the design of multiplex reactions and SBE-PCR primers, guidelines from Chaitanya L. published applying the HIrixPlex-S approach were followed [36]. Specifically, 10 μL of extracted and purified gDNA was used in the multiplex PCR reaction, for which there were distinct concentrations for each sample under investigation as a result of degradation. A Verity 96-Well Fast thermal cycler (Thermo Scientific^®^ purchased at Thermo Scientific Inc.—Via G. B. Tiepolo, 18, 20900 Monza, Italy) was used for all amplification reactions.

The amplified PCR products were purified in order to remove the excess of unincorporated primers in the reaction by enzymatic method using Exonuclease-I (Thermo Scientific^®^ purchased at Thermo Scientific Inc.—Via G. B. Tiepolo, 18, 20900 Monza, Italy).

Following purification, the SBE reaction was set up: after the assembly of the primers located immediately beside the target position, the extension allows a fluorescent ddNTP to be linked to the variable site [44].

Next, the extended products were purified by the enzymatic method again, but this time with alkaline shrimp phosphatase (SAP) to remove excess reaction oligonucleotides and prevent them from producing aberrant fluorescence signals during the detection step. In the end, the products of the second purification step were examined with an ABI 310 HID gene capillary sequencer with POP-4 polymer on a 47 cm-long capillary.

For allele calling and analysis of the results, the Gene Mapper ID-X v1.1 software program was used.

The genotype data obtained by the two multiplex PCR assays were entered in the tool provided by the internet site https://HIrisPlex.erasmusmc.nl (last accessed on 17 February 2023) to generate individual prediction probabilities for eye, hair, and skin colour categories [36].

As an additional analysis control, the victim’s iris, hair, and skin colour were subjectively and objectively determined for all 20 skeletal remains analysed by four independent laboratory operators.

The HIrisPlex-S system allows free evaluation of analytical results in the web interface of https://hirisplex.erasmusmc.nl (last accessed on 17 February 2023). This approach provides a colour prediction of the three phenotypic traits based on genotypic outcomes, associating a probabilistic threshold of accuracy. Initially, the EVC prediction models started to estimate only iris colouration using the IrisPlex approach [43], which achieved a prediction accuracy expressed in the area under the receiver operating curve (AUC) of 0.94 for “blue eye colour”, 0.74 for “intermediate eye colour”, and 0.95 for “brown eye colour”. Following that, with the HIrisPlex model, it was possible to evaluate both eye colour and hair colour at the same time [45]. Using the latter model—of predicting eye and hair colour—it obtains an AUC performance of 0.93 for “red colour”, 0.81 for “blond”, 0.74 for “brown”, and 0.86 for “black”. Finally, the HIrisPlex-S system is developed, which adds skin colour assessment to the power of prediction, realising an AUC performance of 0.83 for “very light”, 0.76 for “pale”, 0.78 for “intermediate”, 0.98 for “dark”, and 0.99 for “dark to black” [46].

For the definition of the categories to which the colour shades of the eyes, hair, and skin colour of individual cases belonged, four examiners were involved in order to classify the colour subcategory (e.g., “pale” or “very light”) from the photographic material available. The results were consistent for about 89% of photographic evaluations. As expected, the evaluation of the pictures showed the highest inconstancy in identifying the intermediate eye and skin colour categories, for which all four examiners provided opposing descriptions.

This limitation in the prediction of intermediate colour categories is attributed to a database that can still be refined and extended to the greatest number of pigmentation combinations of all three phenotypic traits under evaluation.

By enriching the database in this way, there will certainly be an increase in the prediction power and accuracy of the method. An actual improvement in the phenotypic evaluation of forensic samples, in this particular case, will certainly be given by an increase in SNPs describing each trait in terms of colourations.

## 3. Results

Results of the assay showed an overall prediction accuracy of 91.6%, 90.4%, and 91.2%, respectively, for iris, hair, and skin colour, at the 0.7 prediction probability threshold.

**Figure 2:**
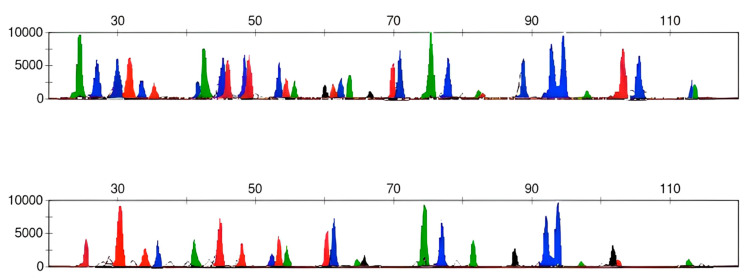
HIrisPlex-S SNP electropherogram obtained from sample I1. The electropherogram shows the amplified SNPs in the same order as those reported in Figure 3. Peaks show the different DNA bases, pointed using the following colour code: green (adenine), black (cytosine), blue (guanine), and red (timine).

Although DNA degradation was observed in the genetic profiles achieved by the STR analysis, the overall PCR amplification of the selected SNPs was successful for both the HIrisPlex and HIrisPlex-S assays. Figure 2 shows an example of an SNP profile achieved after the analysis of the sample identified as I1.

**Figure 3:**
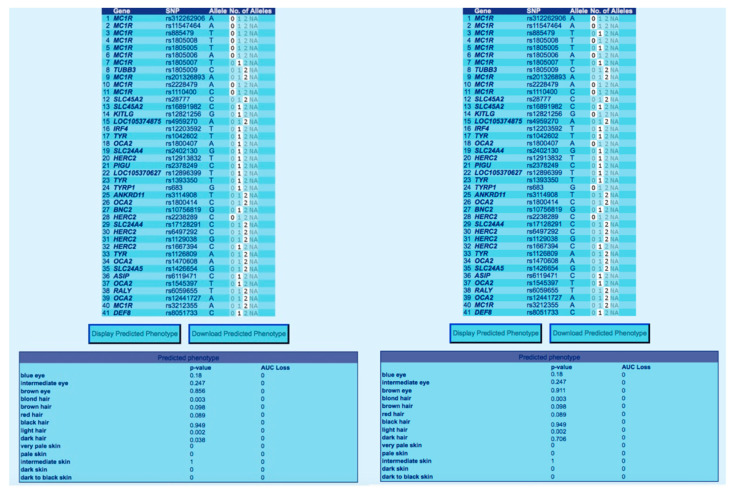
Phenotype and trait prediction shown by samples I7, shown on the left, and I10, shown on the right. Charts taken from entering data in the tool available on the website https://HIrisPlex.erasmusmc.nl (last accessed on 17 February 2023). In the figure, “0” indicates if the input allele is not present; “1” indicates the presence of heterozygosity; and “2” shows the presence of the homozygous input allele; “NA” shows missing SNP.

Among the tested samples, only two turned out to have inconclusive results as compared to the HIrisPlex-S database. These results matched the somatic features shown by the pictures of these specific subjects, but, as documented, these samples showed an intermediate eye and hair colour. Figure 3 shows the prediction values for samples I7 and I10.

Moreover, these specimens were particularly interesting because they were collected from two corpses showing the worst conservation status and the longest post-mortem interval (PMI), respectively, of 7 months and 30 years prior to DNA extraction. These samples showed the highest level of DNA degradation among those tested in the present work.

The evaluation of the STR profiles achieved from the analysis of these samples allowed to identify the loss of larger fragments used in commercial STR kits (greater than 150–170 bp), together with allelic dropout.

## 4. Discussion

Here we present an experimental application in forensic cases of the DNA-based method HIrisPlex-S for the simultaneous prediction of eye, hair, and skin colour.

The applied multiplex genotyping system was previously designed to deal with degraded DNA and was based on a genotyping technology that relies on equipment widely used by the forensic community.

In order to test the HIrisPlex-S assay in forensic cases, the multiplex was performed on a total of 20 different samples, chosen for the assessment, thorough cases in which the biological material was particularly affected. To avoid DNA contamination, bone samples were cleansed chemically, using diluted bleach, and irradiated with UV light for 30 min prior to DNA analysis.

Results showed an overall prediction accuracy of 91.6%, 90.4%, and 91.2%, respectively, for iris, hair, and skin colour, at the 0.7 threshold, with only 2 inconclusive results (samples I7 and I10).

The application of SNaPshot-SBE technology has the advantage of preserving the DNA template and optimising data availability [47]. However, the requirement to increase the robustness and accuracy of the phenotyping method and thus the development of an increased gene panel will result in an increase in the number of loci amplified in multiplex reactions. In contrast, by doing so, it increases the chances of nonspecific bindings, the occurrence of allelic dropouts, and inevitably the preferential amplification of smaller products [47]. In addition, designing primers for similar target fragment lengths involves similar annealing temperatures, so this step could be extremely limiting [48]. For all these reflections, it might therefore be a future prospect to evaluate a Next Generation Sequencing (NGS) approach [49,50,51].

Past works [37,52] and our experience showed that the assay performs successfully in possibly degraded DNA from bodies remains of various age and storage conditions [45]. However, also under such a design, the possibility of allelic dropouts and drop-ins, which have been described as typical phenomena associated with the analysis of low template DNA samples, cannot be eliminated completely [53]. This is to be taken into account, although the cases here analysed were in a much better state of preservation, with PMI being much more recent than the findings in the literature. The incorrect calls that occur by applying the method used in this article may derive from the problem described above. However, the difficulties in the phenotypic determination of those chromatic characters that can vary over time must also be taken into consideration (e.g., a subject with blond hair that turns brown during growth).

The SNaPshot test, as applied here, shows several limitations, among which the most important is related to the limited number of DNA variants that can be evaluated for each test. This implies carrying out several tests with the relative consumption of a larger quantity of DNA. This limitation can be overcome by using massively parallel sequencing (MPS), which has already been applied in other studies with good results [54,55].

While many purport the usefulness of FDP in this regard, its probabilistic nature as well as its ability to disclose information about an individual that may be considered private raise a range of ethical and social concerns [56]. Currently, there are only three European countries that have adopted specific laws in favour of the FDP (the Netherlands, Germany, and Slovakia), although all have limitations in the application of the method. However, in most European countries, even in the absence of specific rules, FDP can be practiced according to the direction of general laws. In the United States, there is no federal law that regulates phenotyping, as there is extreme regulatory variability between states [57,58,59]. One dilemma about the FDP is the judicial applicability of the assay and its application in criminal and civil matters. Indeed, FDP estimates of individual appearance are probabilistic and not deterministic: FDP can create a most likely appearance but cannot generate a perfect likeness of the person. The penal legislative system is based on probabilities close to certainty, unlike civil law, which is based on a probabilistic preponderance. This opens up doubts about the possibility of using the FDP in any context. Actually, it is not necessary to admit FDP in court because once the investigative phase (which can certainly benefit from the FDP) is over, it is quite simple to conduct a standard DNA genotyping and report a match with stronger probabilities. In this way, FDP must only be used for investigative purposes and should never be used as evidence at trial. Genetic diseases are clearly excluded from the phenotyping technique starting with DNA, as it is believed that the use of this data in a forensic context would violate privacy in an unacceptable way [60].

## 5. Conclusions

The results demonstrate the reliability of the HIrisPlex-S genotyping system also in highly degraded DNA samples isolated from bone samples.

These findings may encourage the application of the multiplex for the prediction of eye, hair, and skin traits from DNA in forensic cases, particularly in cases where no other identification method can be applied or where chromatic information can support an anthropological investigation. In fact, the FDP shows a very broad spectrum of applications in the forensic field, both in terms of identification based on traces and cadaveric identification in multiple fields (e.g., missing persons, human trafficking, and mass disasters).

In the future, the implementation of DNA markers related to skin chromatism and their addition to the multiplex system would prove fundamental to improving the prediction accuracies of the “pale” and “intermediate” categories, which with the described model are detected with a much lower accuracy compared to the “dark” and “dark black” skin colour categories [46].

In addition, the future application of this method to a wider case study, even forensic, will allow for the identification of new SNPs for these traits and other visible traits, as well as the development of new prediction models, as already suggested [55]. Definitely, DNA prediction of EVC will become more widely used in genetic studies of human remains in evolutionary, anthropological, and forensic investigations. This preliminary study aims to widen the case studies by combining the cases already selected and further cases of cadavers with different PMI and storage conditions in order to increase the experience in the field. A stronger awareness of the possibilities of this method could lead to its systematic use in forensic cases dealing with unidentifiable corpses, either through the classic genetic method or as a support for classical anthropological analysis.

## Figures and Tables

**Table 1:** Bone samples tested in the present work.

Identified Corpses	Bone Sample	Corpse Preservation
I1	Femur	Skeletonized and burnt
I2	Femur	Highly decomposed
I3	Femur	Burnt
I4	Tibia	Burnt
I5	Tibia	Burnt
I6	Femur	Burnt
I7	Femur	Skeletonized
I8	Tibia	Highly decomposed
I9	Tibia	Highly decomposed
I10	Tibia	Skeletonized
I11	Femur	Highly decomposed
I12	Femur	Highly decomposed
I13	Femur	Skeletonized
I14	Femur	Highly decomposed
I15	Femur	Highly decomposed
I16	Femur	Highly decomposed
I17	Tibia	Highly decomposed
I18	Femur	Highly decomposed
I19	Femur	Skeletonized
I20	Femur	Skeletonized

## Data Availability

All the data used for the article are in the availability of the corresponding author.
